# Incidence and risk of regorafenib-induced hepatotoxicity

**DOI:** 10.18632/oncotarget.21106

**Published:** 2017-09-20

**Authors:** Bin Zhao, Hong Zhao

**Affiliations:** ^1^ The Second Affiliated Hospital and Yuying Children’s Hospital, Wenzhou Medical University, Wenzhou, 325027, China; ^2^ Department of Medical Oncology, The Third Affiliated Hospital of Harbin Medical University, Harbin, 150081, China

**Keywords:** regorafenib, cancer, hepatotoxicity, adverse event

## Abstract

Regorafenib, an oral multi-kinase inhibitor, has been approved for the treatments of several malignancies. Unlike traditional cytotoxic chemotherapeutic agents, regorafenib therapy often induces a distinct profile of adverse events (AEs) including hepatotoxicity. Here we conducted an up-to-date meta-analysis to assess the incidence and risk of regorafenib related hepatic toxicities. PubMed and Embase database were reviewed from inception to June 2017 for relevant trials. Eligible studies include subjects with solid tumors treated with 160 mg of regorafenib daily during the first three week of each four-week cycle, and adequate safety data reporting the elevation of aspartate transaminase (AST), alanine aminotransferase (ALT), alkaline phosphatase (ALP) and bilirubin. Statistical analyses were conducted to calculate the summary incidence and relative risk (RR). A total of 2,213 subjects from 14 trials were included. The incidences of regorafenib-associated all-grade and high-grade hepatotoxicity were: bilirubin elevation: 23% and 5%; AST elevation: 32% and 6%; ALT elevation: 27% and 5%; ALP elevation: 31% and 2%. Regorafenib-treated subjects had a significant increased risk of all-grade (RR *=* 3.10; 95% CI, 2.22–4.34) and high-grade (RR = 1.74; 95% CI, 1.09–2.80) bilirubin elevation; all-grade (RR = 1.51; 95% CI, 1.13–2.00) and high-grade (RR = 1.79; 95% CI, 1.00–3.22) AST elevation; all-grade (RR *=* 1.82; 95% CI, 1.25–2.64) and high-grade (RR = 3.07; 95% CI, 1.30–7.22) ALT elevation; and all-grade (RR = 2.11; 95% CI, 1.01–4.40) ALP elevation. Our results suggest that regorafenib is associated with an increased risk of hepatic toxicities. Hepatotoxicity examination at regular intervals should be advised to clinicians.

## INTRODUCTION

Multi-targeted vascular endothelial growth factor receptor (VEGFR) tyrosine kinase inhibitors (TKIs) have emerged as an important type of anticancer agents. Regorafenib, a novel oral VEGFR TKI, has a distinct molecular target profile and more potent pharmacological activity than sorafenib in pre-clinical investigations [1]. It can inhibit the activity of angiogenic, stromal and oncogenic tyrosine kinases by targeting VEGFR 1, 2 and 3, tyrosine protein kinase receptor Ret, platelet-derived growth factor beta, basic fibroblast growth factor receptor-1, tyrosine-protein kinase TIE-2, proto-oncogene RAF-1, c-KIT, BRAF, and p38 MAP kinase [1, 2]. Currently, regorafenib has been approved by the United States Food and Drug Administration (FDA) for the treatment of advanced gastrointestinal stromal tumor (GIST) [3], metastatic colorectal cancer (CRC) [4], and recently, advanced hepatocellular carcinoma (HCC) [5].

Compared with traditional cytotoxic chemotherapeutic agents, VEGF-targeted TKIs, such as regorafenib, sunitinib and sorafenib are associated with a distinct profile of adverse events (AEs) [6–8]. Previous studies have showed an increased risk of developing hypertension [9], hand-foot skin reaction [10], hematologic toxicities [11, 12], and arterial thromboembolism [13] in patients treated with VEGF-TKIs. In addition, the significant risk of hepatic AE associated with TKI has been reported [14, 15]. It has shown that the incidence of all-grade hepatotoxicity of TKI ranged from 11% (gefitinib) to over 50% (Pazopanib). As for high-grade hepatic AE, the frequencies vary from 1% to 12% [14].

Liver dysfunction is often associated with various symptoms, accordingly a number of biomarkers for cancer therapy induced hepatotoxicity have been identified in the past several decades. Although albumin concentration and prothrombin time (PT) were often used to assess liver function. However, some conditions like heart failure, nephrosis and chronic inflammatory conditions also cause the hypoalbuminemia which can be present in cancer patients. Similarly, it could be difficult to classify patients who is on warfarin, heparin or direct thrombin inhibitors which prolonged PT. In this study, hepatic adverse effects mainly manifest as asymptomatic increase of bilirubin, aspartate transaminase (AST), alanine aminotransferase (ALT) and alkaline phosphatase (ALP).

Fatal adverse events caused by hepatic failure/dysfunction associated with regorafenib-treatment have been reported in several randomized controlled trials (RCTs) such as GRID [16], INTEGRATE [17] and REGOSARC [18]. In addition, the risk for serious hepatotoxicity with regorafenib is believed to be so high that FDA ordered the inclusion of extra labels, the so-called “black box warnings”, to indicate the increased risk of liver injury when patients were treated with regorafenib. However, there has been no systematic attempt to evaluate the overall risk of hepatic toxicities induced by regorafenib. Currently, regorafenib is being investigated in several types of tumors and an increase in the application of regorafenib could be expected in the near future. Accordingly, here we conducted a meta-analysis of available clinical studies to determine the overall incidence and relative risk of developing hepatic AEs in patients treated with regorafenib.

## RESULTS

### Search results

**Figure 1:**
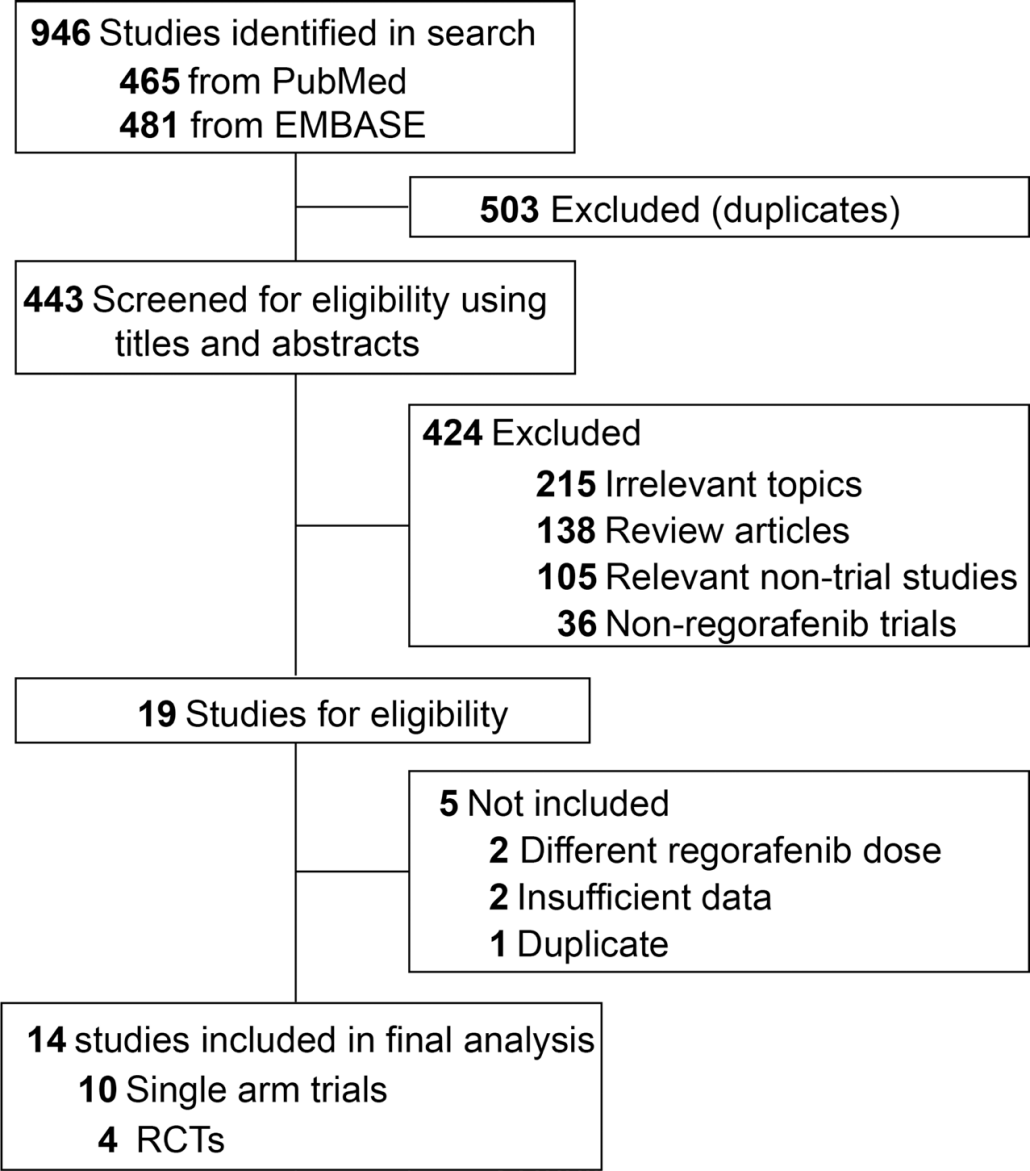
Flow-chart diagram of selected trials included in this meta-analysis

A total of 946 potentially relevant studies were identified by the initial search strategy, including 465 articles on regorafenib from PubMed and 481 papers on regorafenib from Embase database. 503 studies were removed because of duplications. 424 articles were further excluded because they did not satisfy the inclusion criteria. When carefully reviewed the full texts of the remaining 19 potentially eligible papers, 5 more were not included because of insufficient data (*n* = 2) [16, 18], different dose of regorafenib (*n* = 2) [19, 20] and duplication (*n* = 1) [21]. A total of 14 trials were selected for the final analysis. 10 studies were single arm trials [22–31], the other 4 were RCTs [17, 32–34]. A flow chart showing the study selection was presented in Figure 1.

### Study quality

All included phase III trials involved randomized treatment allocation [32–34]. Of the rest 11 trials, 10 trials were single-arm trials [22–31], INTEGRATE was double-blind RCT. For quality analysis purposes, we calculated the incidence in randomized versus non-randomized trials (phase III versus non-phase III). We found no statistically significant difference between subgroups (Data not shown).

### Population characteristics

A total of 2,213 subjects were included in this meta-analysis (regorafenib: 1,649; control: 564). 1,428 subjects had colorectal cancer (regorafenib: 1,107; control: 321) from 9 trials. 603 patients had hepatocellular carcinoma (regorafenib: 410; control: 193) from 2 trials. 147 subjects had gastric cancer (regorafenib: 97; control: 50) from 1 trial. 20 patients had gastrointestinal stromal tumor (regorafenib: 20; control: 0) from 1 trial. The schedule and dose of regorafenib for all trials were 160 mg once daily orally for the first 21 days of each 28-day cycle, the currently FDA-recommended dose until disease progression or unacceptable toxicity. The median treatment ranged from 1.7 months to 9.3 months. The clinic-pathological characteristics of eligible studies were summarized in Table 1. The numbers of all-grade and high-grade hepatic AEs for each trial were presented in Table 2. It was noted that not all trials consistently reported the four hepatic adverse events of our interest.

**Table 1:** Baseline characteristics of the clinical trials included in this study

Author	Region	Year	Underlying malignancy	Follow-up, median (range), month	No. of patients	Median age (range), year	Gender (male/female)	ECOG PS (0/1/2)	Treatment duration, median (range), month	Median OS (95% CI), month	Median PFS (95% CI), month
Li [32]	Asia	2015	CRC	7.4 (4.3–12.2)	136 68	58 (50–66) 56 (49–62)	85/51 33/35	35/101/0 15/53/0	2.4 (1.6–5.3) 1.6 (1.1–1.6)	8.8 (7.3–9.8) 6.3 (4.8–7.6)	3.2 (2.0–3.7) 1.7 (1.6–1.8)
Grothey [33]	Globe	2013	CRC	NR	500 253	61 (54–67) 61 (54–68)	311/194 153/102	265/240/0 146/109/0	1.7 (1.4–3.7) 1.6 (1.3–1.7)	NR NR	1.9 (1.6–3.9) 1.7 (1.4–1.9)
Pavlakis [17]	Globe	2016	GC	17.1 (14.6–19.4)	97 50	63 (33–81) 62 (32–85)	78/19 40/10	41/56/0 21/29/0	1.8 (1.4–2.0) 0.9 (0.9–1.0)	5.8 (4.4–6.8) 4.5 (3.4–5.2)	2.6 (1.8–3.1) 0.9 (0.9–0.9)
Bruix [34]	Globe	2017	HCC	7.0 (3.7–12.6)	379 194	64 (54–71) 62 (55–68)	333/46 171/23	247/132/0 130/64/0	3.6 (1.6–7.6) 1.9 (1.4–3.9)	10.6 (9.1–12.1) 7.8 (6.3–8.8)	3.1 (2.8–4.2) 1.5 (1.4–1.6)
Argiles [22]	Globe	2015	CRC	NR	53	61 (32–80)	28/26	35/19/0	7.7 (0.1–19.5)	NR	8.5 (7.4–11.3)
Kollar [23]	UK	2014	GIST	12.6	20	68 (45–87)	13/7	18/2*	9.3 (0.1–15.3)	12.2	9.4
Sueda [24]	Japan	2016	CRC	5.5	23	59 (37–83)	12/11	10/13/0	2.3 (0.1–14.7)	5.8 (3.7–11.7)	3.0 (1.6–4.5)
Masuishi [25]	Japan	2017	CRC	6.5	146	NR	90/56	135/11*	NR	6.7 (5.8–7.6)	2.1 (1.8–2.5)
Del Prete [26]	Italy	2017	CRC	NR	136	57 (31–79)	92/44	104/32*	3.5	8.9	2.8
Zanwar [27]	India	2016	CRC	NR	23	50	12/11	2/15/6	3.8	NR	NR
Bruix [28]	Globe	2013	HCC	NR	36	61 (40–76)	32/4	28/8/0	4.9 (0.5–25.8)	13.8 (9.3–18.3)	4.3 (2.9–13.1)
Lam [29]	Hong Kong	2016	CRC	6.4	45	63 (45–80)	32/13	41/4*	3.0 (1.0–16.0)	7.6 (4.2–11.1)	3.9 (3.3–4.5)
Schultheis [30]	German	2013	CRC	NR	45	65 (18–80)	27/18	27/16/0	3.6 (0.1–11.5)	NR	4.0 (1.5–11.3)
Sunakawa [31]	Japan	2013	Solid tumor	NR	15	59 (34–68)	11/4	12/3/0	2.1 (0.9–20.1)	NR	3.7 (1.9–12.4)

Abbreviations: CRC, colorectal cancer; GC, gastric cancer; GIST, gastrointestinal stromal tumor; HCC, hepatocellular carcinoma; PFS, progress-free survival; OS, overall survival. NR, not reported; ECOG PS, European cooperative oncology group performance status; *, ECOG 0-1/ECOG 2.

**Table 2:** Number of events reported in every trial included in this study

Author	Year	Underlying malignancy	No. of patients	Events of bilirubin elevation		Events of AST elevation		Events of ALT elevation		Event of ALP elevation		CTCAE
All-grade	High-grade	All-grade	High-grade	All-grade	High-grade	All-grade	High-grade
Li [32]	2015	CRC	136 68	50 5	9 1	32 6	8 0	32 5	9 0	3 1	0 1	4.0
Grothey [33]	2013	CRC	500 253	100 24	38 16	35 10	12 3	27 5	10 0	32 8	11 4	3.0
Pavlakis [17]	2016	GC	97 50	NR NR	NR NR	NR NR	9 0	NR NR	8 3	NR NR	NR NR	4.0
Bruix [34]	2017	HCC	374 193	70 7	25 4	48 15	19 10	29 8	8 2	NR NR	NR NR	4.03
Argiles [22]	2015	CRC	53	NR	NR	12	3	NR	NR	NR	NR	NR
Kollar [23]	2014	GIST	20	2	1	NR	NR	NR	NR	NR	NR	4.0
Sueda [24]	2016	CRC	23	8	1	NR	NR	NR	NR	NR	NR	4.0
Masuishi [25]	2017	CRC	146	70	11	107	19	77	14	NR	NR	4.0
Del Prete [26]	2017	CRC	136	5	0	5	3	NR	NR	NR	NR	4.03
Zanwar [27]	2016	CRC	23	4	1	NR	NR	NR	NR	NR	NR	4.03
Bruix [28]	2013	HCC	36	4	2	NR	NR	NR	NR	NR	NR	3.0
Lam [29]	2016	CRC	45	17	1	26	4	15	3	NR	NR	4.0
Schultheis [30]	2013	CRC	45	NR	NR	3	0	4	2	NR	NR	3.0
Sunakawa [31]	2013	Solid tumor	15	NR	NR	8	2	7	2	14	2	3.0

Abbreviations: CTCAE, common terminology criteria for adverse events; AST, aspartate transaminase; ALT, alanine aminotransferase; ALP, alkaline phosphatase; CRC, colorectal cancer; GC, gastric cancer; GIST, gastrointestinal stromal tumor; HCC, hepatocellular carcinoma; NR, not reported.

### Overall incidence of hepatotoxicity

The pooled incidences of all-grade hepatic toxicities were: increased blood bilirubin, 23% (95% CI, 15%–32%); elevated AST, 32% (95% CI, 19%–46%); elevated ALT, 27% (95% CI, 16%–38%) and elevated ALP, 31% (95% CI, 13%–50%). The incidences of high-grade hepatic AEs were: increased blood bilirubin, 5% (95% CI, 2%–8%); elevated AST, 6% (95% CI, 3%–8%); elevated ALT, 5% (95% CI, 3%–7%) and elevated ALP, 2% (95% CI, 1%–3%). The test for heterogeneities were significant for all-grade and high-grade of these four hepatic AEs (*p* < 0.05 or I^2^ > 25%). Accordingly, the random-effects models were used.

### Relative risk of hepatic toxicity events

**Figure 2:**
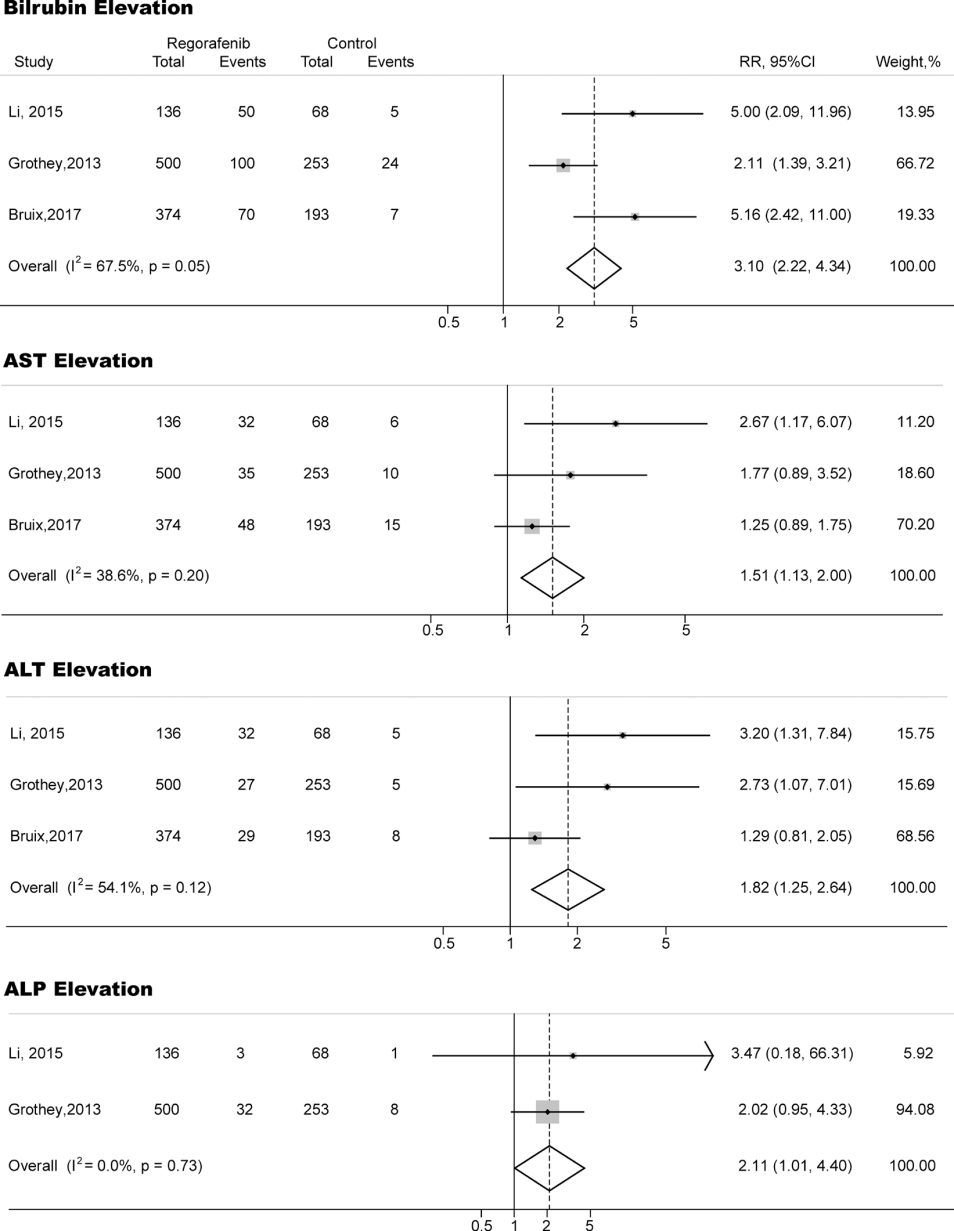
Forest plots of relative risk (RR) of all-grade hepatic toxicities associated with regorafenib versus control The size of squares corresponds to the weight of the trial in the meta-analysis.

**Figure 3:**
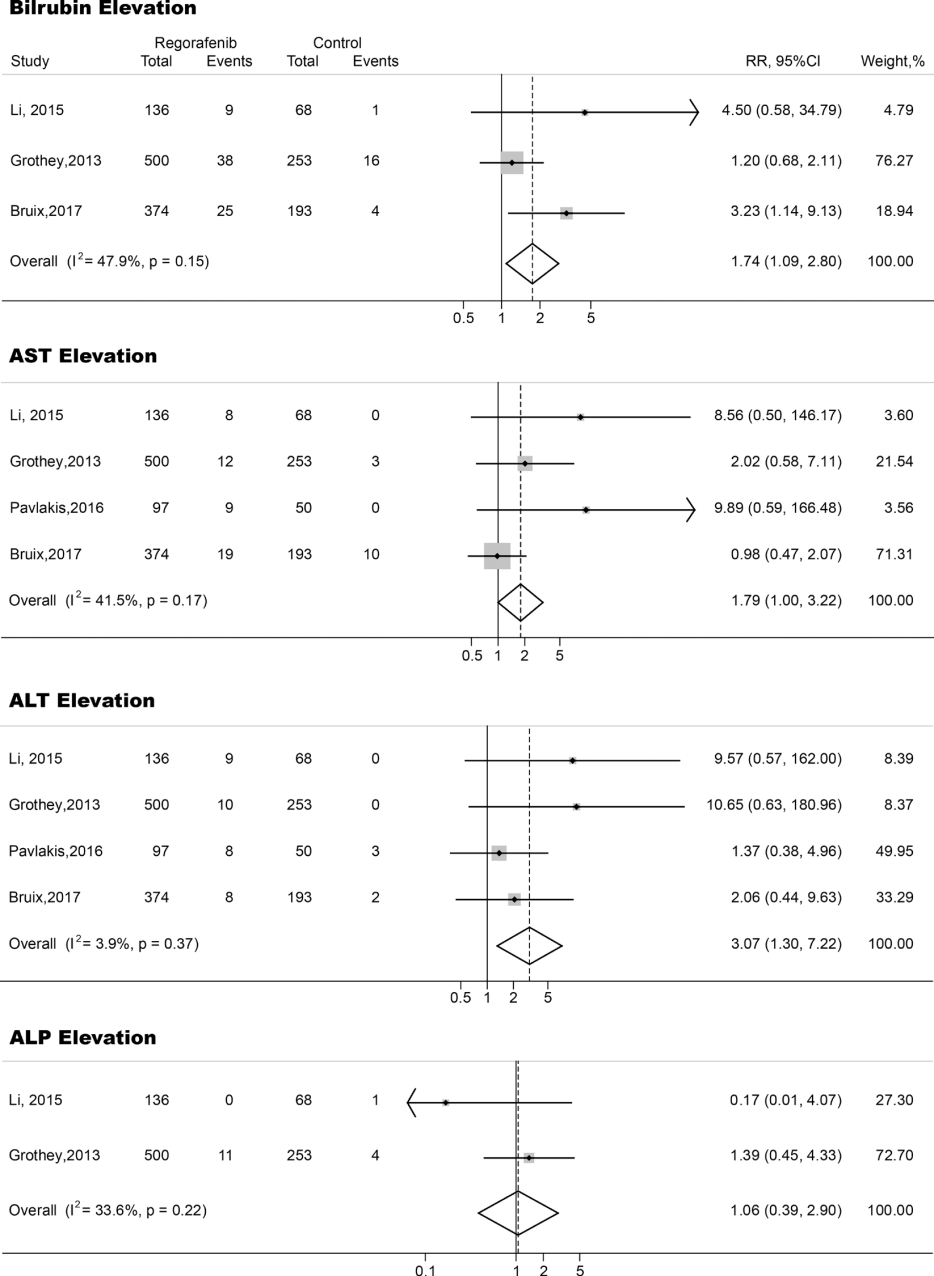
Forest plots of relative risk (RR) of high-grade hepatic toxicities associated with regorafenib versus control The size of squares corresponds to the weight of the trial in the meta-analysis.

A meta-analysis of the RRs and their 95% CIs of both all-grade and high-grade hepatic toxicities was performed on 4 RCTs (3 phase III studies and 1 phase II studies). A total of 1,671 patients were included, 1,107 of them were treated with regorafenib, the rest 564 subjected were treated with placebo. The RRs and their 95% CIs of all-grade elevation of bilirubin, AST, ALT and ALP were 3.10 (95% CI, 2.22–4.34; *p <* 0.001), 1.51(95% CI, 1.13–2.00; *p <* 0.01), 1.82 (95% CI, 1.25–2.64; *p <* 0.001) and 2.11 (95% CI, 1.01–4.40; *p <* 0.05), respectively (Figure 2). The relative risk of high-grade elevation of bilirubin, AST, ALT and ALP in subjects treated with regorafenib were 1.74 (95% CI, 1.09–2.80; *p <* 0.01), 1.79 (95% CI, 1.00–3.22; *p <* 0.05), 3.07 (95% CI, 1.30–7.22; *p <* 0.01) and 1.06 (95% CI, 0.39–2.90; *p >* 0.05), respectively (Figure 3).

### Publication bias

We found no evidence of publication bias for RR of both all-grade and high-grade hepatotoxicities by either the Egger or the Begg test (*p* > 0.05).

## DISCUSSION

The exact incidence of drug-induced hepatic injury is difficult to investigate as the number of patients using any specific anti-cancer agent is uncertain. There is no easy examination for its diagnosis and systemic reporting is incomplete [35]. To our knowledge, this is the first meta-analysis focusing specifically on hepatic toxicities associated with regorafenib. Our results revealed the incidence of regorafenib-induced all-grade and high-grade (grade 3 and 4) hepatic toxicities were: bilirubin elevation: 23% and 5%; AST elevation: 32% and 6%; ALT elevation: 27% and 5%; ALP elevation: 31% and 2%. Furthermore, our analysis demonstrated the risk of developing all-grade hepatic toxicities was approximately two-fold higher in patients treated by regorafenib compared to patients in the placebo or controlled arms. High-grade bilirubin, AST and ALT elevations also significantly increased, while high-grade ALP elevations showed a trend for increase for subjects exposed to regorafenib. Although the incidence of life-threatening hepatic failure/dysfunction reported with regorafenib [16–18] was so small that we cannot analyze the pooled data in current study, we believe that careful monitoring of liver function and exclusion of subjects with hepatic impairment may be essential in regorafenib treatment.

Liver is the regulator of chemical homeostasis in the human body and the main site for detoxification of drugs and their metabolites. Accordingly, any potentially toxic metabolite may cause a localized damage. In addition, liver has great capability to regenerative and recovery. However, it is this regenerative capacity that leads to the cytotoxicity from chemotherapy. So liver injury may be an attempting to kill cancerous cells, but generates more problems sometimes when the secondary hepatotoxicity becomes too severe [36]. Interestingly, it has been reported that in most cases hepatic AEs is caused by treatment with multi-kinase inhibitors, especial TKIs [37]. Previous studies have revealed that some TKIs such as imatinib [38] or pazopanib [39] can induce histologic alteration, inflammation, even acute liver failure in cancer patients. Although accumulating preclinical and clinical evidence suggest that TKIs are associated with hepatotoxicity, the underlying mechanism of hepatic AEs remains unclear. Several theories have been proposed during the past two decades. One possible explanation is the generation of reactive metabolites upon metabolism [40, 41]. These highly reactive metabolites may be bind to the cysteine groups of proteins such as cytochrome P450 1A1 and 3A4, and thereby affecting cell function and cell death. On the other hand, shah et al. demonstrates that the multiple signal transduction pathways inhibited/activated during oxidative stress play an important role in drug-induced liver injury [42, 43]. As for regorafenib, pre-clinical studies suggest that uncoupling of oxidative phosphorylation (OXPHOS) and the resulting mitochondrial permeability transition (MPT) induction and adenosine-triphosphate (ATP) shortage contribute to the hepatocyte injury [44]. Now it is generally believed that various molecular mechanisms are involved in the hepatic AEs, and further studies are needed to reveal the exact reasons underlying regorafenib-associated hepatotoxicity.

TKIs have been shown to cause hepatotoxicity. However, the frequency and severity vary among different agents [14, 15]. The discrepancies are partly due to the differences in the mechanisms of action among these agents, the type of underlying malignancies, under reporting, poor follow-up time of exposed patients among trials included in our analysis and other previous studies. Regorafenib has a structure similar to sorafenib differing only in the fluorine on the phenyl ring [2, 45]. However, compared with sorafenib, regorafenib appears to have a higher risk of increased AST, ALT, ALP and bilirubin levels (Table 3). Although the mechanisms underlying this difference have not been completely explained, it cannot rule out that the structural dissimilarity between regorafenib and sorafenib results in the inhibitory effect on UDP-glucuronosyltransferase (UGT) enzymes such as human liver microsomal β-estradiol glucuronidation [46].

**Table 3:** Relative risk of hepatic toxicities with anti-angiogenic agents

	All-grade: Relative risk (95% CI)				High-grade: Relative risk (95% CI)				References
	AST elevation	ALT elevation	ALP elevation	Bilirubin elevation	AST elevation	ALT elevation	ALP elevation	Bilirubin elevation	
Regorafenib	1.51 (1.13–2.00)	1.82 (1.25–2.64)	2.11 (1.01–4.40)	3.10 (2.22–4.34)	1.79 (1.00–3.22)	3.07 (1.30–7.22)	1.06 (0.39–2.90)*	1.74 (1.09–2.80)	Current study
Sorafenib	1.43 (1.04–1.97)	1.53 (1.18–1.99)	1.41 (1.04–1.91)	1.24 (0.98–1.56)*	2.25 (1.38–3.67)	1.31 (0.67–2.56)*	1.27 (0.60–2.72)*	1.64 (0.89–3.02)*	[15]

Abbreviations: AST, aspartate transaminase; ALT, alanine aminotransferase; ALP, alkaline phosphatase; *p > 0.05.

Although the incidence of fetal hepatic AEs reported with regorafenib is quite limited [16–18], there are currently no methods to predict subjects at high risk and therefore careful monitoring the function of liver and exclusion of subjects with minor hepatic injury may be essential in subjects treated with regorafenib. Subjects suspected of having drug-induced hepatic impairment should be examined thoroughly of any other hepatic diseases such as biliary obstruction, non-alcoholic fatty liver disease, viral hepatitis, et al. In addition, any potential hepatotoxic medication or agents inhibiting regorafenib should be considered carefully before use. In fact, guidelines have been provided by the manufacturer for the management of hepatic toxicities by some agents, and their adoption may alleviate the risk of hepatic AEs. In regorafenib, FDA recommended that close monitoring with biweekly liver function enzyme measurement for the first two months of therapy should be conducted.

Here, in spite of our stringent exclusion and inclusion criteria, we still managed to gather more than 2,000 patients in current meta-analysis, even though several phase II or III RCTs were excluded. The most common reason for exclusion was due to lack of reporting of liver AEs. The rigorous criterion provided confidence of great quality data and a better comparison, whereby the risk of hepatotoxicity could be regarded with assurance to be associated with regorafenib. However, there were several limitations and challenges in this analysis. First, this was a meta-analysis conducted at the trial level and no clinicopathological variables at the patient level could be analyzed. Second, elevation of bilirubin, AST, ALT and ALP represented hepatic injury but these characteristics did not have good specificity and sensitivity. However, giving the number of life-threatening hepatic failure/dysfunction was quite small, these tests were the only feasible method with available data. Third, the data of hepatic toxicities were not present in many trials, leading to their exclusion from current study. AEs, not like efficacy outcomes, were rarely predetermined for systematic data collection in clinical trials. Accordingly, results of hepatotoxicity highly depended on the investigators, and might be confounded by other clinicopathological characteristics as well, such as presence of liver metastasis. Forth, the pooled incidence of hepatic toxicities had significant heterogeneities, and this maight be due to the different types of underlying malignancies, small sample size among the included trials. Fifth, different versions of Common Terminology Criteria for Adverse Events (CTCAE) criteria were applied for grading. However, classifications of various hepatic AEs were unchanged across these versions.

In conclusion, our meta-analysis revealed that regorafenib was associated with an increased risk of hepatic toxicities. Clinical doctors should be acknowledged of these potential adverse events and hepatotoxicity monitoring at regular intervals should be conducted.

## MATERIALS AND METHODS

The present study was conducted in compliance with the recommendations of the *Cochrane Handbook for Systematic Reviews of Interventions* and was reported according to the Preferred Reporting Items for Systematic Reviews and Meta-Analyses (PRISMA) statement [47].

### Literature search and study selection

A comprehensive systematic search of PubMed and Embase up to June 2017 was carried out without any language restrictions. The only keyword was regorafenib. Both eligibility and exclusion criteria were pre-specified. To be eligible, published trials had to meet the following criteria: (1) patients with solid tumor; (2) patients assigned to treatment with regorafenib at a dose of 160 mg orally once daily during weeks 1–3 of every 4-week cycle; (3) events rates and/or events and sample size available for all-grade and high-grade hepatic toxicities including bilirubin, AST, ALT and ALP. For incidence study, trials that assigned patients to regorafenib monotherapy were used to define the incidence of hepatic AE associated with regorafenib as a single agent. For relative risk study, we included trials that randomly assigned subjects to either control or regorafenib in addition to the same treatment to avoid potential confounding in the risk of hepatic toxicities. Other publications on the topic, including conference abstract, review articles, basic science papers, editorials, early versions of data later published, articles not dealing with regorafenib were not included (Figure 1). Since recent studies with regorafenib therapy may be unpublished, electronic searches were also conducted using the major international congresses’ proceedings (European Society of Medical Oncology and American Society of Clinical Oncology Annual Meeting). Any discrepancies were settled by discussion and consensus.

### Data extraction

Identified abstracts were collected and full texts of potentially relevant studies were reviewed for the trial design and reporting of hepatic AEs. The following items were extracted from every study: first author’s name, region, year of publication, underlying malignancy, median follow-up, number of patients for analysis, median age, gender, European cooperative oncology group performance status (ECOG PS), median treatment duration, median overall survival, median progression-free survival (Table 1), events of the following adverse events (both all-grade and high-grade): elevation of bilirubin, AST, ALT and ALP (Table 2). All the reviewers discussed and resolved any discrepancies in the extracted information.

### Statistical analysis

The primary analysis investigated the incidence, relative risk (RR) and corresponding 95% confidence intervals (CIs) of all-grade (Grade 1–4) and high-grade (Grade 3 and 4) hepatic AEs in patients treated with regorafenib. To calculate the incidence, the number of subjects receiving regorafenib alone and the number of subjects with hepatic toxicities (both all-grade and high-grade) were extracted from the eligible single-arm and randomized controlled trials. The proportion of patients with hepatotoxicity and 95% CIs was derived from every study. We calculated both RRs and CIs with data extracted only from randomized controlled trials, comparing the incidence of each adverse event in subjects assigned to regorafenib with subjects assigned to control. Statistical heterogeneity between different trials or subgroups was assessed by Cochrane’s Q statistic. The I^2^ statistic was calculated to assess the extent of inconsistency contributable to the heterogeneity across different studies [48]. The assumption of homogeneity was considered invalid for I^2^ > 25% or *p <* 0.05. Summary RRs and incidences were calculated using fixed-effects or random-effects models depending on the heterogeneity of included trials. When substantial heterogeneity was not observed, the pooled estimate calculated based on the fixed effects model was reported by using inverse variance method. When substantial heterogeneity was observed, the pooled estimate calculated based on the random-effects model was reported by using the DerSimonian and Laird method, which considers both between-study and within-study variations [49]. Potential publication bias was assessed by visual inspection of a funnel plot, and also evaluated using the tests of Egger et al. [50] and Begg et al. [51]. Two-sided *p <* 0.05 were considered statistically significant. All analysis was performed using Stata version 12.0 (StataCorp LP, USA).

## References

[1] Wilhelm SM, Dumas J, Adnane L, Lynch M, Carter CA, Schutz G, Thierauch KH, Zopf D (2011). Regorafenib (BAY 73-4506): a new oral multikinase inhibitor of angiogenic, stromal and oncogenic receptor tyrosine kinases with potent preclinical antitumor activity. Int J Cancer.

[2] Strumberg D, Schultheis B (2012). Regorafenib for cancer. Expert Opin Investig Drugs.

[3] Poveda A, Garcia Del Muro X, Lopez-Guerrero JA, Cubedo R, Martinez V, Romero I, Serrano C, Valverde C, Martin-Broto J (2017). GEIS guidelines for gastrointestinal sarcomas (GIST). Cancer Treat Rev.

[4] Nappi A, Berretta M, Romano C, Tafuto S, Cassata A, Casaretti R, Silvestro L, De Divitiis C, Alessandrini L, Fiorica F, Ottaiano A, Nasti G (2017). Metastatic colorectal cancer: role of target therapies and future perspectives. Curr Cancer Drug Targets.

[5] Desai JR, Ochoa S, Prins PA, He AR (2017). Systemic therapy for advanced hepatocellular carcinoma: an update. J Gastrointest Oncol.

[6] Llovet JM, Ricci S, Mazzaferro V, Hilgard P, Gane E, Blanc JF, de Oliveira AC, Santoro A, Raoul JL, Forner A, Schwartz M, Porta C, Zeuzem S (2008). Sorafenib in advanced hepatocellular carcinoma. N Engl J Med.

[7] Iacovelli R, Palazzo A, Procopio G, Santoni M, Trenta P, De Benedetto A, Mezi S, Cortesi E (2014). Incidence and relative risk of hepatic toxicity in patients treated with anti-angiogenic tyrosine kinase inhibitors for malignancy. Br J Clin Pharmacol.

[8] Zhao H, Guo L, Zhao H, Zhao J, Weng H, Zhao B (2015). CXCR4 over-expression and survival in cancer: a system review and meta-analysis. Oncotarget.

[9] Wang Z, Xu J, Nie W, Huang G, Tang J, Guan X (2014). Risk of hypertension with regorafenib in cancer patients: a systematic review and meta-analysis. Eur J Clin Pharmacol.

[10] Belum VR, Wu S, Lacouture ME (2013). Risk of hand-foot skin reaction with the novel multikinase inhibitor regorafenib: a meta-analysis. Invest New Drugs.

[11] Funakoshi T, Latif A, Galsky MD (2013). Risk of hematologic toxicities in cancer patients treated with sunitinib: a systematic review and meta-analysis. Cancer Treat Rev.

[12] Schutz FA, Je Y, Choueiri TK (2011). Hematologic toxicities in cancer patients treated with the multi-tyrosine kinase sorafenib: a meta-analysis of clinical trials. Crit Rev Oncol Hematol.

[13] Abdel-Rahman O, Fouad M (2014). Risk of cardiovascular toxicities in patients with solid tumors treated with sunitinib, axitinib, cediranib or regorafenib: an updated systematic review and comparative meta-analysis. Crit Rev Oncol Hematol.

[14] Teo YL, Ho HK, Chan A (2013). Risk of tyrosine kinase inhibitors-induced hepatotoxicity in cancer patients: a meta-analysis. Cancer Treat Rev.

[15] Ghatalia P, Je Y, Mouallem NE, Nguyen PL, Trinh QD, Sonpavde G, Choueiri TK (2015). Hepatotoxicity with vascular endothelial growth factor receptor tyrosine kinase inhibitors: A meta-analysis of randomized clinical trials. Crit Rev Oncol Hematol.

[16] Demetri GD, Reichardt P, Kang YK, Blay JY, Rutkowski P, Gelderblom H, Hohenberger P, Leahy M, von Mehren M, Joensuu H, Badalamenti G, Blackstein M, Le Cesne A (2013). Efficacy and safety of regorafenib for advanced gastrointestinal stromal tumours after failure of imatinib and sunitinib (GRID): an international, multicentre, randomised, placebo-controlled, phase 3 trial. Lancet.

[17] Pavlakis N, Sjoquist KM, Martin AJ, Tsobanis E, Yip S, Kang YK, Bang YJ, Alcindor T, O’Callaghan CJ, Burnell MJ, Tebbutt NC, Rha SY, Lee J (2016). Regorafenib for the Treatment of Advanced Gastric Cancer (INTEGRATE): A Multinational Placebo-Controlled Phase II Trial. J Clin Oncol.

[18] Mir O, Brodowicz T, Italiano A, Wallet J, Blay JY, Bertucci F, Chevreau C, Piperno-Neumann S, Bompas E, Salas S, Perrin C, Delcambre C, Liegl-Atzwanger B (2016). Safety and efficacy of regorafenib in patients with advanced soft tissue sarcoma (REGOSARC): a randomised, double-blind, placebo-controlled, phase 2 trial. Lancet Oncol.

[19] Osawa H (2017). Response to regorafenib at an initial dose of 120 mg as salvage therapy for metastatic colorectal cancer. Mol Clin Oncol.

[20] Strumberg D, Scheulen ME, Schultheis B, Richly H, Frost A, Buchert M, Christensen O, Jeffers M, Heinig R, Boix O, Mross K (2012). Regorafenib (BAY 73-4506) in advanced colorectal cancer: a phase I study. Br J Cancer.

[21] Yoshino T, Komatsu Y, Yamada Y, Yamazaki K, Tsuji A, Ura T, Grothey A, Van Cutsem E, Wagner A, Cihon F, Hamada Y, Ohtsu A (2015). Randomized phase III trial of regorafenib in metastatic colorectal cancer: analysis of the CORRECT Japanese and non-Japanese subpopulations. Invest New Drugs.

[22] Argiles G, Saunders MP, Rivera F, Sobrero A, Benson A, Guillen Ponce C, Cascinu S, Van Cutsem E, Macpherson IR, Strumberg D, Kohne CH, Zalcberg J, Wagner A (2015). Regorafenib plus modified FOLFOX6 as first-line treatment of metastatic colorectal cancer: A phase II trial. Eur J Cancer.

[23] Kollar A, Maruzzo M, Messiou C, Cartwright E, Miah A, Martin-Liberal J, Thway K, McGrath E, Dunlop A, Khabra K, Seddon B, Dileo P, Linch M (2014). Regorafenib treatment for advanced, refractory gastrointestinal stromal tumor: a report of the UK managed access program. Clin Sarcoma Res.

[24] Sueda T, Sakai D, Kudo T, Sugiura T, Takahashi H, Haraguchi N, Nishimura J, Hata T, Hayashi T, Mizushima T, Doki Y, Mori M, Satoh T (2016). Efficacy and Safety of Regorafenib or TAS-102 in Patients with Metastatic Colorectal Cancer Refractory to Standard Therapies. Anticancer Res.

[25] Masuishi T, Taniguchi H, Hamauchi S, Komori A, Kito Y, Narita Y, Tsushima T, Ishihara M, Todaka A, Tanaka T, Yokota T, Kadowaki S, Machida N (2017). Regorafenib Versus Trifluridine/Tipiracil for Refractory Metastatic Colorectal Cancer: A Retrospective Comparison. Clin Colorectal Cancer.

[26] Del Prete S, Cennamo G, Leo L, Montella L, Vincenzi B, Biglietto M, Andreozzi F, Prudente A, Iodice P, Savastano C, Nappi A, Montesarchio V, Addeo R (2017). Adherence and safety of regorafenib for patients with metastatic colorectal cancer: observational real-life study. Future Oncol.

[27] Zanwar S, Ostwal V, Gupta S, Sirohi B, Toshniwal A, Shetty N, Banavali S (2016). Toxicity and early outcomes of regorafenib in multiply pre-treated metastatic colorectal adenocarcinoma-experience from a tertiary cancer centre in India. Ann Transl Med.

[28] Bruix J, Tak WY, Gasbarrini A, Santoro A, Colombo M, Lim HY, Mazzaferro V, Wiest R, Reig M, Wagner A, Bolondi L (2013). Regorafenib as second-line therapy for intermediate or advanced hepatocellular carcinoma: multicentre, open-label, phase II safety study. Eur J Cancer.

[29] Lam KO, Lee KC, Chiu J, Lee VH, Leung R, Choy TS, Yau T (2016). The real-world use of regorafenib for metastatic colorectal cancer: multicentre analysis of treatment pattern and outcomes in Hong Kong. Postgrad Med J.

[30] Schultheis B, Folprecht G, Kuhlmann J, Ehrenberg R, Hacker UT, Kohne CH, Kornacker M, Boix O, Lettieri J, Krauss J, Fischer R, Hamann S, Strumberg D (2013). Regorafenib in combination with FOLFOX or FOLFIRI as first- or second-line treatment of colorectal cancer: results of a multicenter, phase Ib study. Ann Oncol.

[31] Sunakawa Y, Furuse J, Okusaka T, Ikeda M, Nagashima F, Ueno H, Mitsunaga S, Hashizume K, Ito Y, Sasaki Y (2014). Regorafenib in Japanese patients with solid tumors: phase I study of safety, efficacy, and pharmacokinetics. Invest New Drugs.

[32] Li J, Qin S, Xu R, Yau TC, Ma B, Pan H, Xu J, Bai Y, Chi Y, Wang L, Yeh KH, Bi F, Cheng Y (2015). Regorafenib plus best supportive care versus placebo plus best supportive care in Asian patients with previously treated metastatic colorectal cancer (CONCUR): a randomised, double-blind, placebo-controlled, phase 3 trial. Lancet Oncol.

[33] Grothey A, Van Cutsem E, Sobrero A, Siena S, Falcone A, Ychou M, Humblet Y, Bouche O, Mineur L, Barone C, Adenis A, Tabernero J, Yoshino T (2013). Regorafenib monotherapy for previously treated metastatic colorectal cancer (CORRECT): an international, multicentre, randomised, placebo-controlled, phase 3 trial. Lancet.

[34] Bruix J, Qin S, Merle P, Granito A, Huang YH, Bodoky G, Pracht M, Yokosuka O, Rosmorduc O, Breder V, Gerolami R, Masi G, Ross PJ (2017). Regorafenib for patients with hepatocellular carcinoma who progressed on sorafenib treatment (RESORCE): a randomised, double-blind, placebo-controlled, phase 3 trial. Lancet.

[35] Leise MD, Poterucha JJ, Talwalkar JA (2014). Drug-induced liver injury. Mayo Clin Proc.

[36] Senior JR (2010). Unintended hepatic adverse events associated with cancer chemotherapy. Toxicol Pathol.

[37] Karczmarek-Borowska B, Salek-Zan A (2015). Hepatotoxicity of molecular targeted therapy. Contemp Oncol (Pozn).

[38] Tonyali O, Coskun U, Yildiz R, Karakan T, Demirci U, Akyurek N, Benekli M, Buyukberber S (2010). Imatinib mesylate-induced acute liver failure in a patient with gastrointestinal stromal tumors. Med Oncol.

[39] Klempner SJ, Choueiri TK, Yee E, Doyle LA, Schuppan D, Atkins MB (2012). Severe pazopanib-induced hepatotoxicity: clinical and histologic course in two patients. J Clin Oncol.

[40] Chan EC, New LS, Chua TB, Yap CW, Ho HK, Nelson SD (2012). Interaction of lapatinib with cytochrome P450 3A5. Drug Metab Dispos.

[41] Li X, Kamenecka TM, Cameron MD (2009). Bioactivation of the epidermal growth factor receptor inhibitor gefitinib: implications for pulmonary and hepatic toxicities. Chem Res Toxicol.

[42] Shah RR, Morganroth J, Shah DR (2013). Hepatotoxicity of tyrosine kinase inhibitors: clinical and regulatory perspectives. Drug Saf.

[43] Han D, Shinohara M, Ybanez MD, Saberi B, Kaplowitz N (2010). Signal transduction pathways involved in drug-induced liver injury. Handb Exp Pharmacol.

[44] Weng Z, Luo Y, Yang X, Greenhaw JJ, Li H, Xie L, Mattes WB, Shi Q (2015). Regorafenib impairs mitochondrial functions, activates AMP-activated protein kinase, induces autophagy, and causes rat hepatocyte necrosis. Toxicology.

[45] Yang M, Zhao H, Guo L, Zhang Q, Zhao L, Bai S, Zhang M, Xu S, Wang F, Wang X, Zhao B (2015). Autophagy-based survival prognosis in human colorectal carcinoma. Oncotarget.

[46] Miners JO, Chau N, Rowland A, Burns K, McKinnon RA, Mackenzie PI, Tucker GT, Knights KM, Kichenadasse G (2017). Inhibition of human UDP-glucuronosyltransferase enzymes by lapatinib, pazopanib, regorafenib and sorafenib: Implications for hyperbilirubinemia. Biochem Pharmacol.

[47] Liberati A, Altman DG, Tetzlaff J, Mulrow C, Gotzsche PC, Ioannidis JP, Clarke M, Devereaux PJ, Kleijnen J, Moher D (2009). The PRISMA statement for reporting systematic reviews and meta-analyses of studies that evaluate health care interventions: explanation and elaboration. Plos Med.

[48] Higgins JP, Thompson SG, Deeks JJ, Altman DG (2003). Measuring inconsistency in meta-analyses. BMJ.

[49] DerSimonian R, Laird N (1986). Meta-analysis in clinical trials. Control Clin Trials.

[50] Egger M, Davey Smith G, Schneider M, Minder C (1997). Bias in meta-analysis detected by a simple, graphical test. Bmj.

[51] Begg CB, Mazumdar M (1994). Operating characteristics of a rank correlation test for publication bias. Biometrics.

