# Correction: Incidence and risk of regorafenib-induced hepatotoxicity

**DOI:** 10.18632/oncotarget.26576

**Published:** 2019-01-04

**Authors:** Bin Zhao, Hong Zhao

**Affiliations:** ^1^ The Second Affiliated Hospital and Yuying Children’s Hospital, Wenzhou Medical University, Wenzhou, 325027, China; ^2^ Department of Medical Oncology, The Third Affiliated Hospital of Harbin Medical University, Harbin, 150081, China

**This article has been corrected:** The correct author affiliation information is given below:

**Hong Zhao^1,2^**

Original article: Oncotarget. 2017; 8:84102-84111. https://doi.org/10.18632/oncotarget.21106

